# A106 PATIENTS’ BARRIERS TO OPTIMIZING DIETARY INTERVENTIONS IN INFLAMMATORY BOWEL DISEASE (IBD): A DESCRIPTIVE QUALITATIVE STUDY

**DOI:** 10.1093/jcag/gwae059.106

**Published:** 2025-02-10

**Authors:** V Noejovich, R Verma, P M Miranda, J Szeto, E Verdu, D Armstrong

**Affiliations:** Medicine, McMaster University, Hamilton, ON, Canada; Medicine, McMaster University, Hamilton, ON, Canada; Medicine, McMaster University, Hamilton, ON, Canada; Medicine, McMaster University Faculty of Health Sciences, Hamilton, ON, Canada; Medicine, McMaster University, Hamilton, ON, Canada; Medicine, McMaster University, Hamilton, ON, Canada

## Abstract

**Background:**

Diet is a key influence on symptoms and disease activity in IBD patients, but effective dietary intervention relies on patient adherence, which is often sub-optimal in clinical practice and research. Adherence is shaped by individual barriers, beliefs and behaviours that must be addressed when recommending personalized dietary interventions, but these barriers are not well understood for IBD patients.

**Aims:**

To identify IBD patients’ self-identified barriers to dietary modification and to understand their experiences and expectations for dietary interventions.

**Methods:**

Adult patients with confirmed IBD attending a tertiary care centre IBD Clinic were invited to join heterogeneous focus group sessions with 2-6 participants or individual interviews moderated by a psychologist over a video communication platform (Zoom). Participants completed a demographics survey (REDCap). Audio files for all sessions were transcribed, de-identified and reviewed for accuracy by two reviewers, followed by thematic analysis (NVIVO).

**Results:**

Between May 2022 and May 2023, 47 IBD patients were enrolled; 38 took part in 11 focus groups and 9 in individual interviews. Most participants (mean age 42 yrs; 60% female) were Caucasian (87%); 42% had a self-reported history of mental health disorders. Mean IBD duration was 16 yrs (0.5–44 yrs); 73% were in remission, and 68% had Crohn’s disease. Participants identified multiple barriers, which were consolidated using thematic analysis into 4 key themes, which encompassed 12 subthemes and related barriers (Table). Some patients (n= 30) reported adjusting their diet when diagnosed with IBD without following a specific diet, while some reported that diet was not discussed with their doctors (n=30).

**Conclusions:**

IBD patients report multiple barriers to modifying their diet, highlighting the need to integrate specialized dietary advice into their healthcare. These findings could be used to develop screening questionnaires to guide personalized dietary interventions that address individuals’ barriers to dietary adoption

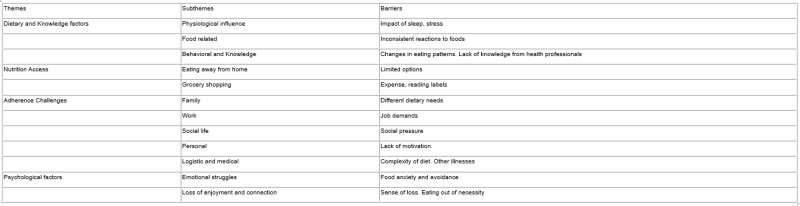

**Funding Agencies:**

None

